# Are pediatric surgery fellowship websites ready for the changing paradigms in the virtual interview era?

**DOI:** 10.1007/s44186-023-00104-w

**Published:** 2023-01-25

**Authors:** Marla A. Sacks, Young Do Jeong, Yomara S. Mendez, Asra Hashmi, Andrei Radulescu, Edward P. Tagge, Jason O. Robertson, Faraz A. Khan

**Affiliations:** 1 Division of Pediatric Surgery, Loma Linda University Children’s Hospital, Loma Linda University Medical Center, 11175 Campus Street, CP21111, Loma Linda, CA 92350 USA; 2 School of Medicine, Loma Linda University, Loma Linda, CA USA; 3 Department of Plastic Surgery, Loma Linda University Health, Loma Linda, CA USA; 4 Department of Pediatric Surgery, Cleveland Clinic Children’s Hospital, Cleveland, OH USA

**Keywords:** Pediatric surgery fellowship, Applicant, Website

## Abstract

**Purpose:**

With the COVID-19 pandemic, in-person fellowship interviews were curtailed, leading candidates to seek information from other resources. Our main purposes were (1) to determine what information recent participants in the match needed to evaluate programs and (2) to assess which of these were available online.

**Methods:**

A focus group of ten recent graduates/applicants identified information that was important in choosing a fellowship program. In August 2020 and December 2021, websites belonging to the American Pediatric Surgical Association (APSA) and individual programs were assessed.

**Results:**

Recent applicants identified 55 pieces of information considered important to their decision making. Of 57 pediatric surgery fellowships, 98% were listed on APSA’s website. Program descriptions on APSA’s website listed on average 60% of program information desired by applicants. All listed fellowship director, accreditation status, faculty list, and current fellow(s). Other descriptors frequently noted were alumni (95%), graduate’s board performance (83%), ECMO exposure (77%), and curriculum (70%). Information desired but less frequently available were fellow case logs (63%), trauma center designation (53%), burn center designation (40%), research opportunities (30%), candidate interview assistance (25%), and supplemental fellowships (12%). There were 7% of program descriptions that were not updated for at least a year.

**Conclusions:**

APSA and individual program websites were complimentary. Websites often lacked data that applicants sought to inform their rank list. To best adapt to the evolving virtual interview paradigm, we suggest reporting key information on a central APSA website with more nuanced information available via links to program specific websites.

**Supplementary Information:**

The online version contains supplementary material available at 10.1007/s44186-023-00104-w.

## Introduction

Recent data show that more than 80% of graduating general surgery residents plan to enter a fellowship [1]. Only the most competitive graduates compete for highly coveted spots in pediatric surgery. Pediatric surgery is frequently considered the most competitive surgical sub-specialty following general surgery training [2–5]. There are 43–47 positions in the United States and Canada for aspiring pediatric surgery trainees each year [6, 7], and the match success rate averaged over the last 5 years is only 50% [6]. More recently though, the number of applicants vying for these positions has decreased from 96 and 93 applicants in 2017 and 2018 to 68 applicants in 2021 [7, 8]. The most recent match year (2021) had the highest success rate and lowest total number of applicants since 2012 [9, 10]. While this could be cyclical and not reflective of an actual trend, it still bears consideration.

Prior to mandatory remote interviews developed in response to the COVID-19 pandemic, a survey of general surgery residents applying to fellowship showed that 62% could have eliminated interviewing at potential programs if their websites had been more useful and informative [11]. Most pediatric surgery fellowship applicants reported that on-site interviews were still a vital component to finding the “best fit;” however, starting in 2020, classic in-person interview processes were curtailed [12]. Therefore, applicants were required to seek relevant data points on various training programs through alternative means, including information available online. While other surgical specialties, such as plastic surgery [13], otolaryngology [14], craniofacial surgery [15], aesthetic surgery [16], hand surgery [17], vascular surgery [18], and cardiothoracic surgery [19, 20] have studied website details available to assist their applicants, there has been no such review for pediatric surgery fellowship programs to our knowledge. This is a critical area to focus attention, as websites have been frequently cited as the most important source of information influencing program application and ranking [18, 21].

Our purposes for this study were to (1) identify what specific program characteristics applicants considered important to make informed decisions for their rank list, (2) determine what proportion of the above-mentioned program characteristics were readily available to applicants online. We additionally analyzed how information available on pediatric surgery fellowship program websites compared to that available on websites for other specialty fellowships.

## Methods

### Programs

Institutional Review Board approval was waived because neither participants nor institutions were identified in this study. Pediatric surgery fellowship programs and frequency of match participation were identified using websites for the National Resident Matching Program (NRMP), Accreditation Council for Graduate Medical Education (ACGME), the Fellowship and Residency Electronic Interactive Database Access (FREIDA), and the Electronic Residency Application Service (ERAS) [8, 22–24].

### Criteria selection

The senior author first reviewed the published data and developed a list of criteria that potential applicants would possibly consider relevant while deciding the rank order list. A focus group of seven pediatric surgery fellows and recent fellowship graduates was convened (see Appendix I, focus group demographics). The list of criteria was then distributed to all other members of the focus group for their input. All suggestions were included in the final tally of criteria queried on the individual program/APSA websites.

### Data collection

Two independent reviewers (M.A.S. and Y.D.J.) searched for the 55 criteria identified by the focus group by querying the APSA and individual program websites. During the course of our study, the APSA website underwent structural changes with updated program descriptions. Therefore, the program characteristics available online via the APSA website were reviewed twice: in August 2020 and again in December 2021 to check for any interim updates. When programs included links to the Graduate Medical Education (GME) office and affiliate hospitals or surgery departments, these links were followed, as well. A third reviewer (F.A.K.) assessed 10 random programs with < 5% disagreement in data collection noted.

### Outcome variables

The primary outcome was the online availability of information regarding the 55 program characteristics deemed important by the focus group to assist with developing a well-informed rank list during the pediatric surgery fellowship application process. Secondary outcomes included the accessibility of a direct, functional link to the program and a date stamp for the most recent update.

### Statistics

Most data points are descriptive in nature and are reported as mean ± standard deviation or number and percentage. Multiple comparisons were made using one-way ANOVA and continuous data was compared using the student’s *t*-test. *P*-values of less than 0.05 were considered statistically significant.

## Results

### Identification of fellowship information important to prospective applicants

**Fig. 1:**
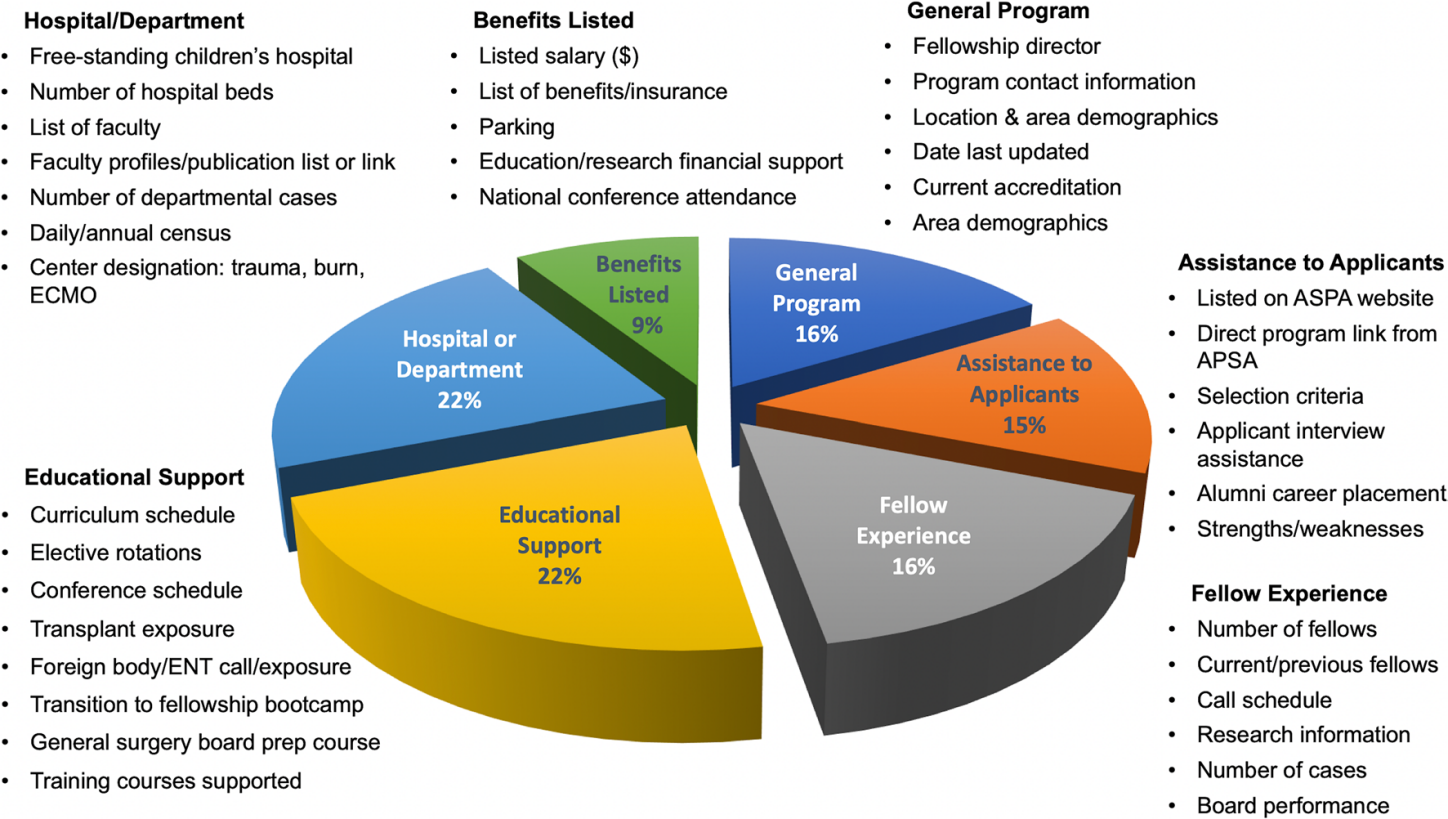
List of all program characteristics identified by the focus group and queried on websites. *NICU* Neonatal Intensive Care Unit, *PICU* Pediatric Intensive Care Unit, *APSA* American Pediatric Surgical Association, *NRMP* National Resident Matching Program, *ENT* ear, nose, and throat, *CRC* Colorectal course, *MIS* minimally invasive surgery, *ECMO* extracorporeal membrane oxygenation

The focus group created a list of 55 discrete program characteristics considered important for making an informed assessment of the individual training program. These 55 program characteristics were organized into six categories for evaluation (Fig. 1).

### Comparison of fellowship data available on APSA and individual program websites

**Fig. 2:**
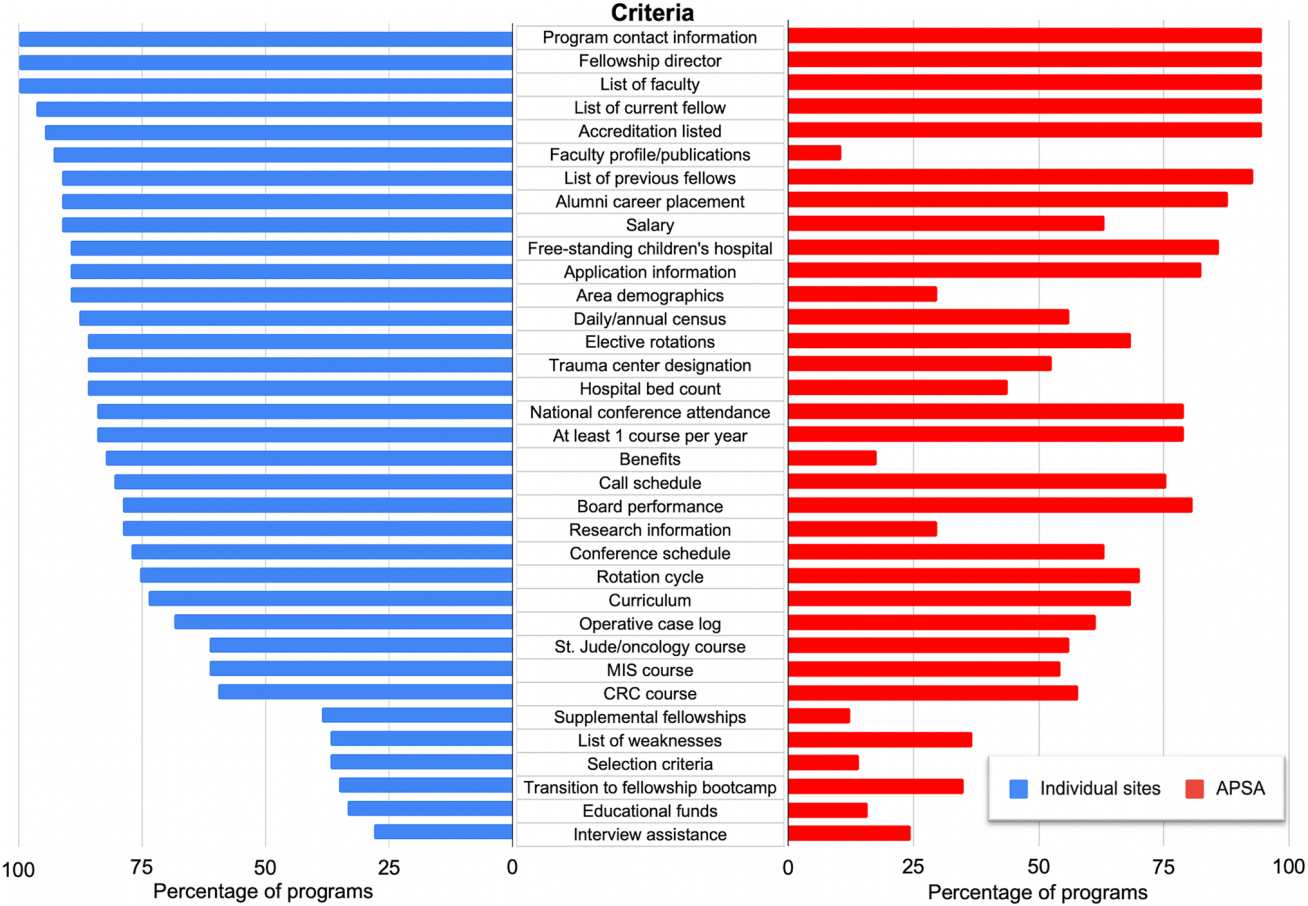
Comparison of individual program websites versus the American Pediatric Surgical Association (APSA) listing program characteristics

Of the 57 pediatric surgery training programs identified, 54 (94.7%) were listed on APSA’s website during the original review. Between the original and follow up reviews, two more programs were added. Program descriptions contained an average of 33 ± 5.5 potential criteria (60%). All webpages listed the fellowship director, accreditation status, staff surgeons, and current fellow. Differences in information available on the APSA website and individual program websites are detailed for several key criteria in Fig. 2.

All programs were grouped by number of fellows and geographic region for comparison. There were no significant differences in the website content available from programs based on number of fellows, department caseloads, hospital type, level I trauma center designation, or designation as a burn or ECMO center (Table 1).

**Table 1:** APSA website program demographic characteristics compared by program type

Characteristic *N* (%)	All programs (*N* = 57)	3 fellows (*N* = 3)	2 fellows (*N* = 38)	1 fellow (*N* = 16)	*p* value
Direct link on APSA website	50 (87.7)	3 (100.0)	34 (89.5)	13 (81.3)	0.421
Stand-alone children’s hospitals	48 (84.2)	3 (100.0)	34 (89.5)	11 (68.8)	0.064
Trauma level designation	26 (45.6)	1 (33.3)	15 (39.5)	10 (62.5)	0.126
Burn center	6 (10.5)	1 (33.3)	3 (7.9)	2 (12.5)	0.602
ECMO program	13 (22.8)	1 (33.3)	10 (26.3)	2 (12.5)	0.273
Fellow cases	41 (71.9)	2 (66.7)	28 (73.7)	11 (68.8)	0.718
Department cases	24 (42.1)	2 (66.7)	16 (42.1)	6 (37.5)	0.759
Salary	41 (71.9)	1 (33.3)	27 (71.0)	13 (81.3)	0.444

*APSA* American Pediatric Surgical Association, *ECMO* Extracorporeal Membrane Oxygenation

### Fellow experience and educational support

When reviewing reporting of the fellow experience and educational support, programs presented a variety of information. Programs reported information about the following characteristics on the APSA website: alumni 54 (95%), whether the hospital classified as a stand-alone children’s hospital 49 (86%), graduate’s board performance 47 (83%), availability of an ECMO program 44 (77%), curriculum 40 (70%), fellow case logs 36 (63%), and level of trauma center designation 30 (53%). Less than half of programs listed burn center designation 23 (40%), available research opportunities 17 (30%), or candidate interview assistance 14 (25%) (Table 2).

**Table 2:** APSA website education details for fellow programs

Characteristics, *N* (%)	All programs (*n* = 57)	2 fellows (*n* = 32)	1 fellow (*n* = 19)	*p*-value
Curriculum	40 (70.2)	22 (38.6)	14 (24.6)	0.774
Special rotations	39 (68.4)	22 (38.6)	11 (19.3)	0.630
Ear, Nose, & Throat	25 (43.9)	19 (33.3)	13 (22.8)	0.729
Urology	41 (71.9)	35 (61.4)	25 (43.9)	0.113
Transplant	27 (47.4)	25 (43.9)	21 (36.8)	0.005*
Conference Schedule	39 (68.4)	22 (38.6)	11 (19.3)	0.630
Research Information	17 (29.8)	11 (19.3)	4 (7.0)	0.489
*Financial Support*				
Interview assistance	14 (24.6)	10 (17.5)	3 (5.3)	0.323
Transition to fellowship	20 (35.1)	12 (21.1)	6 (10.5)	0.609
Colorectal course	33 (57.9)	20 (35.1)	9 (15.8)	0.138
Laparoscopic course	31 (54.4)	18 (31.6)	9 (15.8)	0.492
Oncology course	32 (56.1)	19 (33.3)	10 (17.5)	0.366
National Conference^a^	45 (78.9)	26 (45.6)	14 (24.6)	0.515
At least 1 course or conference per year	45 (78.9)	25 (43.9)	15 (26.3)	0.743
Educational funds	9 (15.8)	5 (8.8)	3 (5.3)	1.000
Accreditation status	55 (95.5)	32 (56.1)	17 (29.8)	0.047*
Supplemental fellowships	7 (12.3)	6 (10.5)	0 (0)	0.037*
Faculty biographies	6 (10.5)	5 (8.8)	1 (1.8)	0.392
Alumni placement	51 (89.5)	30 (52.6)	17 (29.8)	0.348
*Benefits*				
Health insurance	10 (17.5)	6 (10.5)	3 (5.3)	1.000
Area demographics	17 (29.8)	13 (5.3)	3 (5.3)	0.125

^a^American Association of Pediatrics (AAP) or APSA conferences,

^*^p < 0.05 is statistically significant

### General program information, assistance to applicants, and benefits

For each pediatric surgery training program listed on the APSA website, there was a section for listing the current Resident Review Committee (RRC) Accreditation status. The accreditation status was listed for the vast majority of programs on the APSA website 55 (95%). Previous fellows or alumni were listed 54 (95%) and alumni placements after fellowship 51 (90%) were frequently listed. While these are important for convincing fellows they will succeed in the program, other key considerations, such as the presence of pediatric surgery subspeciality fellowships (PSSF) 7 (12%), area demographics 17 (30%), and health insurance offerings 10 (18%) were not well listed on the APSA website; however, they were more frequently noted on individual program websites (Table 3).

**Table 3:** Comparing Information by websites

Characteristics, *N* (%)	APSA (*n* = 57)	Individual (*n* = 57)	*p*-value
Supplemental Fellowships	7 (12.3)	22 (38.6)	< 0.0005*
Faculty biographies	6 (10.5)	53 (93.0)	< 0.0005*
Research Information	17 (29.8)	45 (78.9)	< 0.0005*
*Benefits*			
Health Insurance	10 (17.5)	47 (82.4)	< 0.0005*
Parking	0	34 (59.6)	< 0.0005*
Area Demographic	17 (29.8)	51 (89.5)	< 0.0005*

^*^p < 0.05 is statistically significant

### Keeping the APSA website updated

Almost all of the 56 programs listed on APSA had a notation of when the most recent update had taken place (98.2%). While most programs 49 (89%) on the APSA website were listed as most recently updated in February 2020, only 4 (7%) of programs had been updated in the last 12 months at the time of the review in April 2021. After the APSA website changed vendors, another review in December 2021 revealed only 34 (60.7%) had been updated during the calendar year 2021, including the two programs that were added to the APSA website during that time.

### Comparing pediatric surgery to other surgical subspecialties

The APSA website [25] outscored other surgical subspecialties comparing their recent website reviews with overall median score of 80%. Other high scorers included cardiothoracic (71%) [19], craniofacial (50%) [15], and plastics (46%) [13] (see Appendix II).

## Discussion

Our paper identified a total of 55 program characteristics that recent applicants to pediatric surgery fellowship deemed critical to making an informed assessment of the individual training program. While some of these program characteristics were readily available online, there was substantial heterogeneity among programs. There remains considerable room for improvement to ensure that accurate and relevant information is made available to pediatric surgery fellowship applicants in the era of virtual interviews.

During the fellowship match, both applicants and programs try to find the best fit, hopefully matching applicants to fellowships that they consider desirable and allowing programs to find trainees with characteristics that are important to them [18, 21]. During the COVID-19 pandemic, interviews switched to a virtual format, and candidates sought most of the program information from online resources, including APSA and individual program websites, to make informed decisions. Given the logistics involved, virtual interviews may become permanent, and it is evident that now, more than ever before, having accurate and updated website information is paramount. According to a recent survey of senior surgery residents applying to PSSF, 62% reported that they could have eliminated certain programs from consideration if the websites had been more useful and informative [11]. Programs with more readily available information on their websites tend to also attract more competitive candidates [16]. While pediatric surgery fellowship programs are constantly evolving, the information provided on their websites has, unfortunately, trailed behind.

There has been a recent decline in the total number of applicants seeking fellowship training in pediatric surgery [6, 9, 10, 26, 27]. While this decrease may be transient, it is imperative that pediatric surgery fellowship programs provide up-to-date information to benefit not only prospective candidates but the programs themselves. Better informed candidates with a solid understanding of the strengths and weakness of each program and whether programs align with their individual career/training goals are more likely to arrive at a best fit match for both parties.

The American Pediatric Surgeons Association (APSA) has an informative website for applicants; however, at the time of the initial review in August 2020, which corresponded to the start of the application cycle for the year, most program data had not been updated in the preceding 6 months [25]. This implies that the applicants for that interview cycle may have referred to outdated information. Moreover, some programs did not list the timing of the last website update [25]. While applicants ranked advice from mentors 2nd in importance when creating their rank list [12], the real-time program information on updated websites aides candidates with finalizing their informed decision-making [19].

The core characteristics of the program, namely accreditation status, case exposure, and alumni job placement were amongst the most important for applicants. Beres et al. [12] published a survey in 2011 that asked pediatric surgery applicants to rank 18 criteria in descending order of importance when organizing their rank list. The diversity of cases ranked first and the total number of cases ranked third [12]; however, only 63% of programs listed the case load of the previous fellows and only 46% listed the departmental case load on the APSA website. In our review, 51 (90%) listed post-fellowship career placement. Our data suggest that programs should strive to make the number/diversity of cases and post-graduation employment location of previous fellows available, as a testament of the clinical experience and ability of previous fellows to obtain meaningful post-graduation job opportunities.

While the main focus of clinical fellowship is to become a competent pediatric surgeon, in a study of pediatric surgery fellowship applicants, 55% of successful applicants had dedicated research time before or during residency [2]. Only 17 (30%) of programs on the APSA website listed research opportunities compared to 45 (79%) on individual program websites. While most programs do not offer or support dedicated research time during the clinical fellowship, most fellows are still expected to complete research or quality improvement projects. Applicants value academic prestige, which stands 6^th^ out of 18 in order of importance when formulating a rank list [12, 18, 20]. Scientific output along with clinical strength of the department drives the perceived prestige of an individual program. Therefore, programs would benefit from highlighting institutional research endeavors to attract fellowship applicants.

It is also helpful for applicants to know about where they (and their families) will be spending their training years. Applicants ranked spousal input in 9th place when developing their rank list [12]. It is helpful for each program to provide information on domestic considerations, such as housing assistance, local attractions, and city demographics. For example, 38% of vascular surgery fellowship websites listed domestic considerations [18]. In our review, these domestic considerations appear to be mostly absent on the APSA website (available for only 30% of programs); however, 90% of the programs individual websites had this content.

Accurate and updated websites are very helpful to prospective candidates and may attract some of the strongest candidates to consider programs they may not have initially considered the best fit [16]. At a minimum, programs should provide information for a standardized checklist on the ASPA website with additional details available through a functional, updated hyperlink. Interestingly, programs associated with top-ranked medical schools historically have websites which are maintained with less content; however, program reputation is not an excuse for overlooking the need to update the information [17]. While almost all pediatric surgery programs noted the time of most recent update on the APSA website, only 7% had been updated between February 2020 and April 2021, the first year of the COVID Pandemic. Even during a subsequent review in December 2021, after the start of the next application cycle, only 61% of the programs posted updates during the preceding year. Understandably, websites cannot be updated continuously; however, it is vital to have yearly updates corresponding to the start of application seasons to provide applicants with the most pertinent updated information.

One may ask: *how does the information available to pediatric surgery fellowship applicants stand against other surgical subspecialties?* The online data was matched to information published by other surgical subspecialty website reviews. While there is still work to be done, we should commend the APSA website for serving as the central repository for this information and containing details on 60% of characteristics of interest. This compares favorably to other surgical subspecialty societies that have websites containing much less.

There are several limitations in this study. First, the program characteristics deemed important for making an informed decision on where to rank a program may not be generalizable to the entire cohort of applicants and wider scale surveys of the applicants will be needed to make sure accurate and timely information is made available to the applicants. Second, these data was compared to other surgical subspeciality website reviews for benchmarking; however, some criteria are specific to pediatric surgery thereby making a comparison from other subspecialties challenging. Third, this review does not account for other resources that may be available to an applicant, as programs may directly send applicants additional information or they may have alternative resources, such as Instagram^©^, LinkedIn^©^, or Twitter^©^, which are not captured here.

Due to the intense competition and near 100% program fill rate, there may be a perceived minimal incentive to maintain informative up-to-date websites for pediatric surgery fellowships; however, individual programs should see this as an opportunity to attract the best fit applicants. With the changes in application paradigms and applicant rate falling to the lowest since 2012, we should see this as an indicator of change and an opportunity to adapt to the changes in the pediatric surgery fellowship application process.

## Conclusion

As applicants rely on the APSA website as an invaluable resource for Pediatric Surgery training programs in the era of virtual interviews, the information posted becomes increasingly important but limited by its accuracy. We recommend that the APSA page listing all participating programs should have a standardized checklist with suggested information, and, additionally, provide a functional hyperlink to the individual program sites for supplementary information. The program descriptions on APSA should be updated at least yearly, ideally, prior to each application season. Pediatric surgery training programs should also adapt to this evolving paradigm, updating their detailed individual websites to assist future trainees in one of the most competitive general surgical subspecialty matches.

## Supplementary Information

Below is the link to the electronic supplementary material.

Supplementary file1 (DOCX 13 KB)

Supplementary file2 (DOCX 24 KB)

## Data Availability

The datasets generated during and/or analyzed during the current study are available from the corresponding author on reasonable request.

## Abbreviations

APSA: American Pediatric Surgery Association
NRMP: National Resident Matching Program
ACGME: Accreditation Council for Graduate Medical Education
FREIDA: Fellowship and Residency Electronic Interactive Database Access
ERAS: Electronic Residency Application Service
ECMO: Extracorporeal membrane oxygenation
PSSF: Pediatric Surgery Subspeciality Fellowships
GME: Graduate Medical Education
CRC: Colorectal course
MIS: Minimally invasive surgery
