# Supplementary material for: Are pediatric surgery fellowship websites ready for the changing paradigms in the virtual interview era?
Source: Global Surg Educ. 2023 Jan 25;2(1):27. doi: 10.1007/s44186-023-00104-w (PMC9874179; doi:10.1007/s44186-023-00104-w)
Supplement: Supplementary file 1 — Supplementary file1 (DOCX 13 KB) [file 44186_2023_104_MOESM1_ESM.docx]

**Appendix I: Focus group member demographics**

| **Member** | **Gender** | **Position** | **PGY* from residency** | **Practice type** |
| --- | --- | --- | --- | --- |
| 1 | Male | Attending | 4 | Academic |
| 2 | Male | Attending | 4 | Academic |
| 3 | Male | Attending | 3 | Academic |
| 4 | Male | Attending | 3 | Academic |
| 5 | Male | Fellow | 2 | Academic |
| 6 | Female | Fellow | 1 | Academic |
| 7 | Male | Fellow | 1 | Academic |

*PGY: post-graduate year
