# Supplementary material for: Are pediatric surgery fellowship websites ready for the changing paradigms in the virtual interview era?
Source: Global Surg Educ. 2023 Jan 25;2(1):27. doi: 10.1007/s44186-023-00104-w (PMC9874179; doi:10.1007/s44186-023-00104-w)
Supplement: Supplementary file 2 — Supplementary file2 (DOCX 24 KB) [file 44186_2023_104_MOESM2_ESM.docx]

**Appendix II. Comparison of surgical specialty fellowship website content (%)**

|  | APSA^25^ | Plastics^13^ | Cranio-facial^15^ | Aesthetic^16^ | Vascular^18^ | Cardio-thoracic^19^ | Thoracic^20^ |
| --- | --- | --- | --- | --- | --- | --- | --- |
| Program description | 100 | 43 | 95.8 | 85.7 | 97.8 | 58 | 75.4 |
| Contact Information | 100 | 74 | - | - | 76.4 | 89 | 69.2 |
| Current  fellow | 100 | 64 | 41.7 | 32.1 | 41.6 | 77 | 41.5 |
| Current faculty | 100 | 88 | 83.3 | 17.9 | 89.9 | 100 | 56.9 |
| Operative experience | 63.2 | 10 | 75 | 35.7 | 21.3 | 65 | 23.1 |
| Didactics or curriculum | 72.2 | 61 | 58.3 | 28.6 | 68.5 | 69 | 53.8 |
| Call  schedule | 79.6 | 40 | 29.2 | - | 66.3 | 81 | 16.9 |
| Alumni  listing | 98.2 | 30 | - | - | 32.6 | - | 24.6 |
| Research opportunities | 29.8 | 38 | 70.8 | 35.7 | 57.3 | 77 | 40 |
| Salary & Benefits | 66.7 | 55 | 33.3 | 0 | 32.6 | 73 | 32.3 |
| National meeting | 78.9 | 48 | 41.7 | 7.1 | 20.2 | 23 | - |
| Board performance | 82.5 | - | - | - | 4.5 | 5 | 7.7 |
| Local information | 29.8 | 39 | 25.2 | - | 38.2 | 46 | 20.0 |
| **Median** | 79.6 | 45.5 | 50.0 | 30.4 | 41.6 | 71 | 36.2 |
